# Supplementary material for: A comparative study on the physical fitness of college students from different grades and majors in Jiangxi province
Source: PLoS One. 2024 Oct 16;19(10):e0308483. doi: 10.1371/journal.pone.0308483 (PMC11482695; doi:10.1371/journal.pone.0308483)
Supplement: Attachment — Submitted filename: MS- Jianzhong Sun --revised version.docx [file pone.0308483.s003.docx]

Comparative study on physical fitness of different grades and majors for college studentsof Jiangxi province

Jianzhong Sun^1,3^, Chan Lin^1,3^, Lei Wang^2^, Cunjian Bi^1,3^, Bin Qiao^2*^

^1^School of Physical Education, Chizhou University, Chizhou, 247000, China;

^2^Basci Teaching Department, Chizhou Vocational and Technical College, Chizhou, 247000, China;

^3^Sports Health Promotion Center, Chizhou University, Chizhou, 247000, China;

*Corresponding author: Bin Qiao, QB13305663823@126.com

**Abstract**

**Objective:**To determine the extent to which age and major impact the physical health of college students, numerous studies have been conducted on the physical health of children and adolescents.It is postulated that there are significant disparities in physical health among college students of different grades and majors. The objective of this study was to demonstrate the existence of these disparities and to conduct a comprehensive analysis and discussion on them.

**Methods:** A sample of 8772(2404 boys and 6368 girls)Chinese college students from freshman to junior years, aged 17-22,including 12 different majors in four colleges, were recruited in Jiangxi Province. All seven physical fitness indicators (body mass index (BMI), forced vital capacity,50-m dashes，standing long jump, sit and reach, upper body muscle strength, endurance runs)were conducted for every boys and girls student. One-way ANOVA and LSD tests were conducted to compare the physical fitness scores of different grades in the same major. Independent sample *t-*tests were used to compare the differences in every physical fitness indicator for different majors. Pearson’s correlations among 12 majors for every grade were conducted to study the significant corrections between the two physical fitness indicators. The body mass index (BMI) and physical fitness indicator (PFI) for college students of different grade were investigated using a nonlinear regression model.

**Results:** The physical fitness of college students is poor today because most scoresare near the passing line (75.3%). Sophomore-year students had lower scores than sophomore-yearstudents across all 12 majors. From freshman to junior year, music (78.01±4.58), English (79.29±5.03), and education (76.26±4.81) majors had the highest scores, respectively, butart major consistently scored the lowest, which were 73.85±6.02, 74.97±5.53, and 72.59±4.84, respectively. Pairwise comparisons revealed more significant differences in individual physical fitness indicators among the three grades in humanities than in sciences. Pearson’s correlations showed significant correlations among seven physical fitness indicators in all three grades. PFI had a parabolic trend with BMI for both boy and girl college students in Jiangxi province.

**Conclusion:** The physical fitness indicators of college students in Jiangxi province significantly differed in grades and majors, showing undesirable phenomena. The physical fitness of senior and humanities major college students is much weaker and needs sufficient attention. The relationship between BMI and PFI presents an inverted “U”-shaped change characteristic. Continued nationwide interventions are needed to promote physical activity and other healthy lifestyle behaviors in China.

**Keywords:** physical fitness, grade, major, body mass index, physical fitness indicators

**Background**

Physical inactivity can have significantly negative impacts on physical fitness, thereby increasing the risk of non-communicable diseases and mortality^1, 2^.In accordance with the latest World Health Organization (WHO) guidelines for physical activity, adults should aim to engage in at least 150-300 minutes of moderate intensity aerobic activity weekly, or 75-150 minutes of high-intensity aerobic activity weekly, or a combination of both moderate and high-intensity activities^3^.Most of Swedish children do not reach the recommended daily activity level^4^.Poor physical fitness leads to a decrease in the body's immune system, making it easier for bacteria and viruses to invade.Physical inactivity is a significant contributor to cardiovascular disease, diabetes, and obesity, which can heighten the risk of developing conditions such as hypertension, hyperlipidemia, osteoporosis, depression, diabetes, obesity, and heart disease, ultimately leading to life-threatening outcomes^5,6^.Being overweight and obesity are also the main manifestations of poor physical fitnessnowadays^7^, and have been condemned as the fourth largest risk factors for death after hypertension, dietary risk, and tobacco use^8^. The latest data show that over 1.3 million people worldwide die from overweight and obesity every year^9^. Poor lifestyle habits, such as physical inactivity and poor diets,also significantly impact physical fitness^10^. Su et al. revealed that total daily physical activity had a significant positive effect on cancer (*P* < 0.05)^6^.

Fitness refers to a series of physical exercises designed to enhance muscle development, improve physical strength, enhance body shape, and improve mental well-being through the use of either bare hands or various equipment. It encompasses five key elements: clear fitness goals, a blend of aerobic and anaerobic activities, regular exercise, a balanced diet, and rest and recovery. According to the inaugural Global Health Index, which evaluated 146 countries and regions, the average score was 46.96. China's score ranges between 68.1, slightly above the global average.^12^.

There is a significant negative correlation between a sedentary lifestyle and physical fitness level (*P* = 0.00, *P* < 0.05) for Indonesianadolescents^13^. Cantarero et al. believed that the health quality of life was related to physical fitness and a sedentary lifestyle^14^. A significant positive relationship between leisure-time physical activity and physical fitness was demonstrated in both females and males aged 20-59 years^15^. The physical fitness of Chinese college students is also not encouraging. According to the results of national physical fitness monitoring in 2020, the physical fitness of Chinese college students continues to decline^1, 16^. The main manifestation is that the body weight gradually increases, while strength and quality show a decreasing trend^17^. Physical fitness was shown to be positively associated with physical activity among adults aged 40 to 79 years in China^18^. Obesity, physical inactivity, and reduced physical fitness contribute to the rise of chronic diseases in China^19^.

Contemporary college students are the future of our country, the hope of our nation, and shoulder the heavy responsibility of China's takeoff. The physical fitness and health of college students are related to the future and destiny of our country. To the best of our knowledge, there is currently no comprehensive research on the physical well-being of college students in Jiangxi Province, China. This study aims to serve as a valuable reference for enhancing the physical fitness of college students in Jiangxi province, China.Months ago, our research team conducted a cross-sectional study on age, sex, and body mass index (BMI) roles in physical fitness for Chinese college students^20, 21^. In this article, the different grades and majors were considered to reveal their impact on the physical fitness of Chinese college students. This can provide a more comprehensive understanding of the influencing factors of contemporary college students' physical fitness, provide guidance for universities to improve and enhance students' physical fitness^22^ and provide data support for national sports educators to formulate policies^23, 24^.

**Methods**

**Data source and participants**

In this study, four colleges and universities were randomly selected as test schools among all colleges and universities in Jiangxi Province, China. Executedaccording to the 2021Chinese National Survey on Students' Constitution and Health (CNSSCH)^25^. This study mainly focuses on the impacts of grades and majors on the physical fitness of college students. Based on a multistage stratified random cluster sampling method, choose three grades from freshman to junior,including 12 majors (4 science majors and 8 humanitiesmajors) from 4 colleges in Jiangxi province (Table 1). Finally,8772 (2809 freshmen, 2904 sophomores, and 3059 juniors) college students were included in this study.

**Table 1**Participants of twelve majorsin three grades of college students from Jiangxi, China.

| **Subject** | **Major** | **Freshman (%)** | **Sophomore (%)** | **Junior (%)** | **Total** |
| --- | --- | --- | --- | --- | --- |
| Sciences | Math | 258 (9.2) | 278 (9.6) | 287 (9.4) | 823 |
|  | Physics | 249 (8.9) | 261 (9.0) | 258 (8.4) | 768 |
|  | Chemistry | 201 (7.2) | 264 (9.1) | 239 (7.8) | 704 |
|  | Biology | 238 (8.5) | 275 (9.5) | 284 (9.3) | 797 |
| Humanities | Chinese | 235 (8.4) | 246 (8.5) | 260 (8.5) | 741 |
|  | English | 262 (9.3) | 255 (8.8) | 249 (8.1) | 766 |
|  | Law | 242 (8.6) | 223 (7.7) | 229 (7.5) | 694 |
|  | History | 226 (8.0) | 223 (7.7) | 235 (7.7) | 684 |
|  | Art | 298 (10.6) | 272 (9.4) | 270 (8.8) | 840 |
|  | Music | 150 (5.3) | 161 (5.5) | 203 (6.6) | 514 |
|  | Education | 174 (6.2) | 181 (6.2) | 227 (7.4) | 582 |
|  | Economics | 276 (9.8) | 265 (9.1) | 318 (10.4) | 859 |
|  | Sum | 2809 (100) | 2904 (100) | 3059 (100) | 8772 |

This study was approved by the human experimental ethics committee of Chizhou University (No. CZ2022YJR03). Written informed consent was obtained from the participants before this research. The names of the college student participants were coded to protect their privacy.

**Instruments**

Testers must undergo rigorous training before the test to ensure safety and accuracy. All tests used the same manufacturer and batch of instruments according to the standard procedures for college students prescribed in the 2021 CNSSCH. Measurement instruments were calibrated everyday morning and at fixed intervals throughout the day (5 times at least). Every physical fitness indicator test was required to be completed at the same fixed time of the day to reduce data deviation caused by different test times. In this study, seven physical fitness indicators were used, which were BMI (calculated by height and weight), forced vital capacity, 50-m dash, standing long jump, sit and reach, upper body muscle strength (pull-ups for boys and one minute sit-ups for girls), and endurance run (800 m for girls and 1000 m for boys).

1. Body mass index (BMI) = weight (kg)/height (cm). Weight and height were

simultaneously measuredto the nearest 0.1 kg and 0.1 cmwith the electronic instrument (TSN200-ST, Tishineng Sports, made in China), respectively. College participants must upright their bodies, look forward, and take off their shoes and hats.

2. Forced vital capacity was measured using the vital capacity meter (TSN200-FH, Tishineng Sports, made in China). Each participant had a new blowing nozzle. During the test, the participants are in a natural standing position, holding the handle of the venturi tube, and trying their best to inhale deeply. Then, they used the mouth piece to slowly exhale until they were unable. The value displayed on the display screen was the vital capacity value. A total of 2 tests were conducted and recorded the maximum value in milliliters without retaining decimals. The interval between the two tests shold not exceed 15 seconds.

3. The 50-m dashes were measured by the test instrument (TSN200-WP, Tishineng Sports, made in China). The college student participants heard the starting signal and ran as fast as possible to the finish line. 50-m dash is only measured once and the time precision is 0.1s.

4. Standing long jump was measured on the specialized long jump mats (TSN200-TY, Tishineng Sports, made in China), which allowed participants to try twice and record the best score. Each college student stands in front of the starting line with both feet shoulder-width apart, jumping with both feet simultaneously. The longest distance between the starting line and the heel of the nearest shoe was recorded after measuring twice.

5. Sit and reach were measured with the instrument model TSN200-TQ, Tishineng Sports, made in China. The participants removed their shoes, straightened their legs, and sat down with their toes against the testing board. College students slowly bent over and straightened their arms to push the ruler as far as possible, the farthest distance was the best score that needed to be recorded after twice trials.

6. Upper body muscle strength, including pull-ups for boys (TSN200-YT, Tishineng Sports, made in China), and oneminute sit-ups for girls (TSN200-YW, Tishineng Sports, made in China). Pull-ups were tested on horizontal bars, using the back hand method. Upper body strength was used to pull the entire body up. Each move required an elbow angle greater than 90 degrees to record. One minute sit-ups recorded the number of sit-ups in 60 seconds. Participants should bend their knees 90 degrees and lie on a soft cushion. Reaching or passing their knees with their elbows is considered a complete sit-up task. Pull-ups and sit-ups were only tested once.

7. Endurance runs including 1000 m for boys and 800 m for girls, were measured using the long distance running instrument (TSN200-CP, Tishineng Sports, made in China). The test was conducted on a standard track of 400 meters, 2.5 laps for boys and 2 laps for girls. Upon hearing the starting signal, participants ran as fast as they could to the finishing line, recording running time accurately to 0.1s.

The test instruments mentioned above are all bluetooth, which could automatically save and upload data in a standard format.

All seven physical fitness indicators mentioned above were converted to Z-scores via SPSS. The physical fitness indicator (PFI) was obtained using these indicators’Z-scores. The Z-scores for the 50-m dash and endurance run (800 m and 1000 m) were reversed, because lower times reflect better performances in these three tests. Therefore, PFI = Z BMI + Z forced vital capacity –Z 50-m dash + Z standing long jump + Z sit and reach +Z upper body muscle strength −Z endurance run.

**Statistical analysis**

The physical fitness record was expressed as the mean±standard deviation. The chi-square test was utilized to assess the disparities in total physical fitness scores among college students across different grades.One-way ANOVA and LSD tests were conducted to compare physical fitness scores with different grades in the same major. Independent sample *t-*tests were used tocompare the differences in every physical fitness indicator for different majors and sexes.

Pearson’s correlations among 12 majors for every grade were conducted to study the significant corrections between every two physical fitness indicators. The BMI and PFI for college students of different grades were investigated using a nonlinear regression model. All statistical analyses mentioned in this study were conducted with SPSS software version 25.0 (SPSS Inc., Chicago, IL, USA).

**Results**

Based on 12 different majors and physical fitness record levels, the number of college students in three grades was statistically analyzed in Table 2. Most college students fell near the passing line (6607), following students who perform good (2069) and fail (53), with the least number of students performing excellent (43). In the excellent record levels, the number of sophomore students (25) exceeded freshmen (13) and juniors (5). Similarly, with good record levels, the number of sophomore students (947) exceeded freshmen (652) and juniors (470). But, in pass and fail record levels, the number of junior students (2559 and 25, respectively) exceeded freshmen (2124 and 20, respectively) and sophomores (1924 and 8, respectively). Economics majors had the most (10) in excellent, but math, history, and art majors had the lowest (1). Physics majors had the most fail (14), while Chinese and education majors had zero fail students.

An analysis of the differences in total physical fitness scores among college students of varying grades revealed significant disparities across all 12 majors, with 10 of them exhibiting extremely significant differences, excluding biology and music majors.

**Table 2** Statistics of the number of college students in different record levels and grades based on 12 majors.

| **Subject** | **Major** | **Excellent** | | | **Good** | | | **Pass** | | | **Fail** | | | **X^2^** | ***P*** |
| --- | --- | --- | --- | --- | --- | --- | --- | --- | --- | --- | --- | --- | --- | --- | --- |
|  |  | A | B | C | A | B | C | A | B | C | A | B | C |  |  |
| Sciences | Maths | 1 | 0 | 0 | 48 | 72 | 35 | 206 | 206 | 247 | 3 | 0 | 5 | 23.36 | 0.001** |
|  | Physics | 1 | 1 | 0 | 42 | 86 | 38 | 204 | 170 | 212 | 2 | 4 | 8 | 35.11 | 0** |
|  | Chemistry | 0 | 2 | 2 | 40 | 99 | 51 | 158 | 163 | 183 | 3 | 0 | 3 | 28.45 | 0** |
|  | Biology | 0 | 6 | 0 | 67 | 84 | 76 | 168 | 184 | 204 | 3 | 1 | 4 | 14.37 | 0.026* |
| Humanities | Chinese | 0 | 2 | 1 | 50 | 71 | 38 | 185 | 173 | 221 | 0 | 0 | 0 | 17.43 | 0.002** |
|  | English | 0 | 4 | 0 | 69 | 113 | 34 | 193 | 138 | 214 | 0 | 0 | 1 | 70.87 | 0** |
|  | Law | 2 | 1 | 0 | 58 | 92 | 22 | 179 | 130 | 207 | 3 | 0 | 0 | 68.89 | 0** |
|  | History | 1 | 0 | 0 | 54 | 78 | 36 | 170 | 145 | 199 | 1 | 0 | 0 | 28.01 | 0** |
|  | Art | 0 | 0 | 1 | 45 | 45 | 15 | 249 | 225 | 251 | 4 | 2 | 3 | 20.25 | 0.003** |
|  | Music | 1 | 3 | 1 | 50 | 41 | 37 | 99 | 117 | 164 | 0 | 0 | 1 | 14.01 | 0.03* |
|  | Education | 3 | 0 | 0 | 49 | 75 | 47 | 122 | 106 | 180 | 0 | 0 | 0 | 28.07 | 0** |
|  | Economics | 4 | 6 | 0 | 80 | 91 | 41 | 191 | 167 | 277 | 1 | 1 | 0 | 50.39 | 0** |
| Sum | | 13 | 25 | 5 | 652 | 947 | 470 | 2124 | 1924 | 2559 | 20 | 8 | 25 |  |  |
|  |  | 43 (0.5%) | | | 2069 (23.6%) | | | 6607 (75.3%) | | | 53 (0.6%) | | |  |  |

Note: A-Freshman, B-Sophomore, C-Junior; *indicates significant difference, **indicates extremely significant difference.

Table 3 shows the mean and standard deviation of the total physical fitness scores for the three grades with different majors. It can be observed that sophomores performed better than freshmen, and juniors were the worst for all 12 majors except e music. In freshman year, music majors had the highest score (78.01±4.58), and the art majors had the lowest score (73.85±6.02). In sophomore year, English majors had the highest score (79.29±5.03), and art majors had the lowest score (74.97±5.53). In junior year, educationmajors had the highest score (76.26±4.81), and art majors had the lowest score (72.59±4.84). The results of the one-way ANOVA analysis and pairwise comparison of LSD showed that there were significant differences (*P*< 0.01) in the total physical fitness scores across all 12 majors in the three grades. Compare with sophomores, freshmen scores showed that the differences in every major were significant (*P*< 0.05 or *P*< 0.01) except for biology and economicsmajors. Compared with juniors, freshmen showed that the differences in every major were significant (*P*< 0.01) except for math, chemistry, Chinese, and biologymajors.There were significant differences (*P*< 0.01) in physicalfitness scores across all 12 majors between sophomores and juniors.

**Table 3** Comparison of total physical fitness test scores with different grades in every major.

| **Major** | **Freshman (A)** | **Sophomore (B)** | **Junior (C)** | **F/P** | **Difference value/P** | | | | | |
| --- | --- | --- | --- | --- | --- | --- | --- | --- | --- | --- |
|  | Mean±SD | Mean±SD | Mean±SD |  | A-B | P | A-C | P | B-C | P |
| Math | 74.62±6.01 | 76.49±4.95 | 74.03±5.81 | 14.71/0.000 | -1.87 | 0.000 | 0.59 | 0.22 | 2.46 | 0.000 |
| Physics | 74.44±5.98 | 76.17±6.58 | 72.88±6.43 | 17.47/0.000 | -1.72 | 0.002 | 1.57 | 0.006 | 3.29 | 0.000 |
| Chemistry | 74.93±5.89 | 78.36±5.67 | 74.45±6.21 | 23.71/0.000 | -3.43 | 0.000 | -0.52 | 0.36 | 2.91 | 0.000 |
| Chinese | 76.01±5.08 | 77.43±5.04 | 75.58±4.59 | 9.69/0.000 | -1.41 | 0.002 | 0.44 | 0.32 | 1.85 | 0.000 |
| English | 77.04±4.59 | 79.29±5.03 | 75.18±4.97 | 48.59/0.000 | -2.26 | 0.000 | 1.85 | 0.000 | 4.11 | 0.000 |
| Law | 76.14±5.96 | 78.22±5.21 | 73.98±5.64 | 35.13/0.000 | -2.08 | 0.000 | 2.16 | 0.000 | 4.24 | 0.000 |
| History | 76.15±5.47 | 77.79±5.09 | 74.52±5.58 | 21.07/0.000 | -1.64 | 0.001 | 1.63 | 0.001 | 3.27 | 0.000 |
| Art | 73.85±6.02 | 74.97±5.53 | 72.59±4.84 | 11.75/0.000 | -1.12 | 0.016 | 1.26 | 0.007 | 2.38 | 0.000 |
| Music | 78.01±4.58 | 76.79±5.51 | 75.37±5.72 | 10.72/0.000 | 1.21 | 0.046 | 2.64 | 0.000 | 1.43 | 0.012 |
| Education | 77.78±4.85 | 78.93±4.39 | 76.26±4.81 | 16.73/0.000 | -1.15 | 0.021 | 1.52 | 0.001 | 2.68 | 0.000 |
| Biology | 76.94±5.49 | 77.37±5.73 | 75.99±5.78 | 4.28/0.014 | -0.43 | 0.390 | 0.94 | 0.059 | 1.37 | 0.004 |
| Economics | 77.46±5.35 | 78.03±5.98 | 74.59±5.57 | 34.33/0.000 | -0.57 | 0.220 | 2.82 | 0.000 | 3.39 | 0.000 |

Further comparisons of the individual physical fitness test scores in different grades were carried out for every major (Table S1 in supporting information). In all 12 majors, readers can find the individual physical fitness test scores for all seven physical fitness indicators from freshmen to sophomores to juniors and the significant differences in *P* values between every two grades. It is worth noting that there are significant differences in the pairwise comparison of the three grades for law, art, biology, music, and economics majors in 50-m dash, educationmajors in standing long jump of, and biologymajor in endurance run.

To compare the difference between the humanities and sciences, independent sample *t*-tests were used in seven physical fitness indicators for different sexes in Table 4. The total score showed that girl students had higher scores (77.27±4.61 and 77.99±4.39) than boy students (71.99±6.37 and 72.25±6.53) in both humanities and sciencesmajors. For boy students, sciences major performed better in BMI, 50-m dash, standing long jump, upper body muscle strength, and endurance run than humanitiesmajor. For girl students, sciencemajors performed better in six physical fitness indicators, except for forced vital capacity, than humanitiesmajors. There were significant differences (*P*< 0.01) between major humanities and sciences in BMI and standing long jumpfor boys, andin BMI, forced vital capacity, 50-m dash, and upper body muscle strengthfor girls.

**Table 4** Comparison of individual and total physical fitness test scores between Humanities and Sciences of the same sex.

| **Sex** | **Physical fitnessindicators** | **Humanities** | **Sciences** | **T-test** | |
| --- | --- | --- | --- | --- | --- |
|  |  | Mean±SD | Mean±SD | F | *P* |
| Boys | BMI | 14.27±1.29 | 14.34±1.24 | 8.73 | 0.003 |
|  | forced vital capacity | 11.87±0.98 | 11.72±1.95 | 1.31 | 0.25 |
|  | 50-m dash | 15.42±1.82 | 15.66±1.89 | 3.75 | 0.053 |
|  | standing long jump | 6.85±0.95 | 6.91±0.88 | 6.91 | 0.009 |
|  | sit and reach | 7.67±1.07 | 7.62±1.05 | 0.73 | 0.39 |
|  | upper body muscle strength | 2.39±2.91 | 2.51±2.89 | 1.81 | 0.18 |
|  | endurance run | 13.35±1.95 | 13.48±2.05 | 0.061 | 0.81 |
|  | Total score | 71.99±6.37 | 72.25±6.53 | 0.16 | 0.69 |
| Girls | BMI | 14.59±1.03 | 14.69±0.92 | 52.69 | 0 |
|  | forced vital capacity | 11.74±1.94 | 11.65±1.78 | 38.06 | 0 |
|  | 50-m dash | 14.19±1.41 | 14.24±1.28 | 6.22 | 0.013 |
|  | standing long jump | 7.28±0.96 | 7.48±0.95 | 0.99 | 0.32 |
|  | sit and reach | 8.30±1.12 | 8.36±0.14 | 1.45 | 0.23 |
|  | upper body muscle strength | 6.82±0.81 | 6.93±0.87 | 9.31 | 0.002 |
|  | endurance run | 14.36±1.56 | 14.65±1.51 | 2.62 | 0.11 |
|  | Total score | 77.27±4.61 | 77.99±4.39 | 7.21 | 0.007 |

Table 5 shows the results of Pearson’s correlations between every two physical fitness indicators in all 12 majors for every grade divided by sex. There were no significant correlations between BMI and forced vital capacity and between forced vital capacity and upper body muscle strength in all three grades for boys. For boy students, there were also no significant correlations between BMI and sit and reach in freshman year; BMI and sit and reach, 50-m dash and sit and reach in sophomore year; and BMI and upper body muscle strength, forced vital capacity and 50-m dash, and forced vital capacity and endurance run in junior year. For girls, there were no significant correlations between BMI and forced vital capacity, BMI and standing long jump, forced vital capacity and 50-m dash, forced vital capacity and upper body muscle strength, forced vital capacity and endurance run, and 50-m dash and sit and reach in freshman year; BMI and forced vital capacity, BMI and upper body muscle strength, forced vital capacity and endurance run, and 50-m dash and sit and reach in sophomore year; and BMI and standing long jump in junior year. Besides the correlations mentioned above, there were statistically significant correlations (*P*< 0.05 or *P*< 0.01) between every two physical fitness indicators used in this study.

**Table 5** Correlations between physical fitness indicators for college studentsin every grade by sex (r-value).

| **Sex** | **Grades** | **A/B** | **A/C** | **A/D** | **A/E** | **A/F** | **A/G** | **B/C** | **B/D** | **B/E** | **B/F** | **B/G** |
| --- | --- | --- | --- | --- | --- | --- | --- | --- | --- | --- | --- | --- |
| Boys | Freshman | 0.28 | 0.12^a^ | 0.13^a^ | 0.056 | 0.16^a^ | 0.18^a^ | 0.17^a^ | 0.083^b^ | 0.21^a^ | -0.024 | 0.11^a^ |
|  | Sophomore | -0.003 | 0.12^a^ | 0.16^a^ | -0.014 | 0.1^a^ | 0.21^a^ | 0.16^a^ | 0.095^a^ | 0.029 | -0.06 | 0.11^a^ |
|  | Junior | -0.004 | 0.16^a^ | 0.15^a^ | 0.12^a^ | 0.047 | 0.14^a^ | 0.069 | 0.1^a^ | 0.071^b^ | 0.069 | 0.037 |
| Girls | Freshman | 0.018 | 0.07^a^ | 0.043 | 0.06^a^ | 0.068^a^ | 0.11^a^ | 0.003 | 0.11^a^ | 0.079^a^ | 0.024 | 0.033 |
|  | Sophomore | 0.001 | 0.048^b^ | 0.052^b^ | 0.078^a^ | 0.039 | 0.088^a^ | 0.05^b^ | 0.11^a^ | 0.04 | 0.071^a^ | 0.018 |
|  | Junior | 0.045^b^ | 0.051^b^ | 0.04 | 0.051^b^ | 0.066^a^ | 0.071^a^ | 0.14^a^ | 0.089^a^ | 0.044^b^ | 0.045^b^ | 0.064^a^ |

Continued.

| **Sex** | **Grades** | **C/D** | **C/E** | **C/F** | **C/G** | **D/E** | **D/F** | **D/G** | **E/F** | **E/G** | **F/G** |
| --- | --- | --- | --- | --- | --- | --- | --- | --- | --- | --- | --- |
| Boys | Freshman | 0.19^a^ | 0.12^a^ | 0.15^a^ | 0.34^a^ | 0.13^a^ | 0.22^a^ | 0.31^a^ | 0.12^a^ | 0.13^a^ | 0.28^a^ |
|  | Sophomore | 0.4^a^ | -0.051 | 0.25^a^ | 0.3^a^ | 0.075^b^ | 0.31^a^ | 0.32^a^ | 0.031 | 0.052 | 0.28^a^ |
|  | Junior | 0.41^a^ | 0.12^a^ | 0.24^a^ | 0.31^a^ | 0.17^a^ | 0.26^a^ | 0.23^a^ | 0.13^a^ | 0.13^a^ | 0.19^a^ |
| Girls | Freshman | 0.43^a^ | 0.031 | 0.18^a^ | 0.36^a^ | 0.16^a^ | 0.15^a^ | 0.34^a^ | 0.14^a^ | 0.074^a^ | 0.17^a^ |
|  | Sophomore | 0.45^a^ | 0.03 | 0.21^a^ | 0.39^a^ | 0.14^a^ | 0.23^a^ | 0.32^a^ | 0.069^a^ | 0.098^a^ | 0.22^a^ |
|  | Junior | 0.44^a^ | 0.12^a^ | 0.26^a^ | 0.37^a^ | 0.18^a^ | 0.28^a^ | 0.32^a^ | 0.14^a^ | 0.14^a^ | 0.27^a^ |

Note: ^a^ < 0.01；^b^ < 0.05.

A, BMI; B, forced vital capacity; C, 50- m dash; D, standing long jump; E, sit and reach; F, upper body muscle strength; G, endurance run.

The relationship between BMI Z-scores and PFI Z-scores for girls(Figure 1) and boys (Figure 2) was estimated using the nonlinear regression model(SPSS: Curve estimation-quadratic model). All figures revealed the inverted U-shaped curves in three different grades for both girls and boys. The results of the nonlinear regression model indicated that higher and lower BMI negativelyaffected PFI for both boys and girlscollege students. It found that, overall, the girl college students had notably higher PFI Z-scores than boys with the same BMI Z-scores. Freshman-year students performed the worst. Sophomore-year students performed better than junior-year students for girls, and the junior-year students slightly outperformed sophomores, with BMI Z-scores ranging from -0.5 to 1.7.

**Figure 1**Linear relationship between BMI Z-scores and PFI Z-scores for girls college studentsin each grade.

**Figure 2**Linear relationship between BMI Z-scores and PFI Z-scores for boys college students in each grade.

**Discussion**

Based on the physical fitness record levels, the proportion of excellent (0.5%) and fail (0.6%) groups were the least. The physical fitness score of most college students fell near the passing line (75.3%)^26^, following students who performed good (23.6%). This suggests that the physical fitness of college students nowadays is poor^16, 22, 27, 28^. Upon investigatingpotential reasons, the overwhelming majority of college students were highly impacted by screen time, obesity, and sedentary, leading to less exercise time^29^. This phenomenongrew even worse during the COVID-19 lockdown period for college students^30^. Kidokoro et al. found that the population health declined for Japanese children and adolescents during the COVID-19 pandemic^31^. This fully proves the necessity and significance of China’s national strategy of public fitness. Moreover, this study found that sophomore-year students performed better than freshman and junior year. This finding was consistent with Cao H., who revealed that sophomores had the best pass rate, following freshmen, juniors and seniors^32^.

Generally speaking, sophomores had better total physical fitness scores than freshmen, and juniors had the lowest values, for all the eleven majors except for music.This trend is in line with the current situation of universities revealed by Song Y.^33^and Hu et al.^34^, where the physical fitness of senior students is poorer than that of lower grade students^26^. On the one hand, most colleges do not offer specialized physical education classes for students in their third and fourth years, no longer ideologically valuing physical sports^35^. On the other hand, senior students feel pressured to graduate or find a job, they do not have time to exercise^36^. It is crucial to cultivate a habit of exercise among college students, which will benefit them for their lifetime^37^. Thus, freshman and sophomore students must proficiently master one or two sportskills^38, 39^, be able to play games, and find joy in playing sports, which is conducive to developing their sport habits. Constructing a healthy China requires the support of people's good exercise habits^40, 41^, especially for college students who are valuable talent and the future of our motherland.

For freshman students, music majors had the highest score (78.01±4.58), and the art majors had the lowest score (73.85±6.02). In sophomore year, English majors had the highest score (79.29±5.03), and art majors had the lowest score (74.97±5.53). Wang Z. conducted a study on the physical health of college students of 11 majors in Lvliang College, but had no total score to compare with this study^42^. In junior year, education majors had the highest score (76.26±4.81), and art majors had the lowest score (72.59±4.84). Both art and physics majors had the worst performance in all three grades in the humanities and sciences, respectively, which urgently require sufficient attention and further investigation. Hu et al. found that there were significant differences among different majors, with agronomy (belonging to the sciences) majors having the best performance and Chinese studies (belonging to the humanities) majorsperforming the worst^34^. Readers can find the ranking of 17 majors in his original text^34^.

There were significant differences (*P*< 0.01) in the total physical fitness scores across the three grades in all 12 majors^16^and most results of pairwise comparisons from freshmen to juniors, which means each college student's lifestyle and exercise habits are all different^42-45^. It is worth mentioning that there were significant differences (*P*< 0.01) in the physical fitness scores across all majors between sophomores and juniors^33, 46^. This conclusion was similar to Song Y., who revealed that seniors had the worst scores^33^. Sophomore-year students had lower values than other students in all 12 majors, which is an important discovery of this article^46^. Shen et al. compared the daily physical activity levels between professional female graduates and non-professional, the different majorsbeing one of the important reasons^43^. Sheng et al. found that most students do not engage in other heavy physical activities after completing physical education classes^16^. Senior students no longer have mandatory physical education classes, resulting in a significant decrease in their chances of engaging in sports.

Pairwise comparisons revealed more significant differences inindividual physical fitness indicators among the three grades in thehumanities (law, art, music, education, and economicsmajor) than in sciences (biologymajor). This suggests great imbalances among individual physical fitness indicators in the humanities, while the physical fitness development of science students is relatively balanced. These results conform to many people's preconception that sciences majors have more physical activity than humanities students’. Ping Y. found no significant differences in academic achievements between humanities and sciencestudents majoring in social sports^47^, which means that the physical fitness development of sports majors in the humanities and sciences is relatively balanced.

There are significant differences (*P*< 0.01) in BMI between humanities and sciences students for both boys and girls, with humanities major having lower BMI values than sciences majors. Zhang et al. found that there wassignificant difference among the humanities, sciences, and arts (*P*< 0.001)^48^. In the standing long jump for boys, science students scored significantly (*P*< 0.01) higher than humanities students^48^. Due to the different majors, humanities students may have fewer physical activities and longer sedentary times than science students during non-physical education classes^49^. There are also significant differences (*P*< 0.01) in forced vital capacity, 50-m dash, and upper body muscle strength between humanities and science majors for only girls. This phenomenon indicates that the physical fitness of girls is more influenced by different majors. For example, among all majors, girls majoring in art tended to be slimmer^48^. This high probability may be related to the difference between liberal humanities and science courses. There are more experimental courses in science and far more opportunities for exercise, while there are more liberal arts and history courses in humanities with longer sedentary hours^49^.

Pearson’s correlations among seven physical fitness indicators in three grades ranged from 0.001 to 0.45. Most r-values showed significant correlations (*P*< 0.05 or *P*< 0.01), suggesting a close relationship existed with each other. There were no significant correlations between many physical fitness indicators for boyjuniors (Table5). This maybe the reason for junior students performing worse than freshmen and sophomores in most majors^33, 46^(see Table 3).

The results of this research revealed that PFI had a parabolic trend with BMI for both boy and girl college students in Jiangxi province. This finding was similar to Bi et al., who found inverted U-shaped curves between BMI Z-scores and PFI Z-scores for the young aged 7-18 in Xinjiang from 1985 to 2014^50^.The results indicated that higher and lower BMI negatively affected the PFI for both boys and girlscollege student^51^. The normal weights and heights can help attain a better PFI. To reach this goal, Jiang et al. suggested universities help students develop proper exercise habits and enhance the ideological awareness of the importance of sports^52^. Girl college students had much higher PFI Z-scores than boys with the same BMI Z-score in this research. This trend consistent with the findings of Bi et al.^50, 53^.

Freshman-year students performed the worst. This maybe related to the just completed college entrance examination and the almost three-month-long summer vacation. Parents or students themselves do not attach importance to fitness. Dai C. believed that freshmen have not developed good lifestyle habits and daily physical exercise habits, mostly focused on the online world for daily attention^54^. Sophomore-year students performed better than junior-year students for girls, but junior-year students slightly outperformed sophomores, with BMI Z-scores ranging from -0.5 to 1.7. Most colleges in China do not have physical education (PE) classes for junior and senior students. Combined with senior internships and job hunting, this exacerbates the lack of time for college students to exercise. The life and behavioral habits of college students, excessive screen time, and master’s entrance examination pressure have contributed to the sharp increase in sedentary time^29, 44, 46, 55, 56^, which might be another reason for the poor physical fitness of advanced college students. Therefore, to improve the physical fitness levels of college students, especially advanced college students, necessary measures must be carried out to control excessive thinness or obesity, with treatment strategies implemented and targeted for college students of different grades and majors.

**Limitations of the Study**

This study presents a comparative analysis of the physical health status of college students in Jiangxi Province from two perspectives: grade and major. However, several limitations must be acknowledged. Firstly, there is a scarcity of research data on the physical condition of college students in China, which hinders the conduct of comparative studies. Secondly, the sample size utilized in this study is relatively small and may not accurately represent the physical health status of college students in Jiangxi Province. To address these limitations, future research should involve more scholars who can conduct in-depth studies on local provincial and municipal universities, as well as multi-school, inter-provincial, and inter-municipal collaborations. This approach will enable a more comprehensive understanding and accurate assessment of the physical health status of college students in Jiangxi Province.

**Conclusions**

The physical fitness of college students is poor today because most scoresare near the passing line (75.3%). Sophomore-year students had lower scores than sophomore-year students across all 12 majors. From freshman to junior year, music (78.01±4.58), English (79.29±5.03), and education (76.26±4.81) majors had the highest scores, respectively, but art major consistently scored the lowest, which were 73.85±6.02, 74.97±5.53, and 72.59±4.84, respectively. Different college grades and majors play important roles in the physical fitness of students in Jiangxi province. This study found that, overall, senior-year and humanities students had much poorer physical fitness. The abnormal BMI negatively affected the PFI for both boy and girlcollege students. Issuingan exercise prescription is an effective way to solve this problem, namely, setting up different sports programs based on the characteristics of college students of different grades and majors so that the intensity of exercise meets the requirements of the course objectives. Further research on these exercise prescriptionsis urgently needed for both whole classes and individual students.

**Author’s contributions**

Conceptualization, Methodology, Software, JS and BQ. Data curation, Investigation, CL and LW. Writing - original draft, LW and CL. Writing—review & editing, JS and BQ. All authors have read and agreed to the published version of the manuscript.

**Funding**

This work was supported by“the Ministry of Education Humanities and Social Sciences Research Youth Project Approval of 2023 (Award No. 23YJC890002)”,“School level scientific research project of Chizhou University in 2022 (Award No.CZ2022YJRC03)”, and “Anhui province 2022 provincial quality engineering project for higher education institutions (Award No. 2022jxms116)”.

**Competing interests**

The authors declare no competing interests.

**Additional information**

The datasets used during the current study cannot be made publicly availableas per ethics approval at Chizhou University. Readers can obtain them fromthe corresponding author on reasonable request.

**References**

1. Yigit S, Akinci B, Ulker EB, Korkmaz DD, Calikoglu BF, Yeldan KI, et al. Physical fitness in patients with diabetic dyslipidemia: Diabetes or dyslipidemia to blame? *European Journal of Preventive Cardiology.*2023; Supplement_1. Doi: 10.1093/eurjpc/zwad125.119.

2. Mcdermott MM, Liu K, Ferrucci L, Tian L, Guralnik JM, Liao Y, et al. Greater sedentary hours and slower walking speed outside the home predict faster declines in functioning and adverse calf muscle changes in peripheral arterial disease. *Journal of the American College of Cardiology.*2011; 57(23):2356-2364.[https://doi.org/10.1016/j.jacc.2010.12.038](https://doi.org/10.1016/j.jacc.2010.12.038).

3. Zenu S, Abebe E, Reshad M,et al.Withdrawal: Most healthy adults in urban centers of Southwest Ethiopia are not meeting the WHO's physical activity recommendations. *Scandinavian journal of medicine & science in sports*. 2023； 33(6):1036. Doi: 10.1111/sms.14333.

4. Seger I, Lundvall S, Eklund A, Jamshidpey A, Takats J, Stlman C, et al. A sustainable swedish school intervention with extra aerobic exercise—its organization and effects on physical fitness and academic achievement. *Sustainability MDPI.*2022; 14(5): 1-32.

5. Birna B W, Bernhard S.Physical activity in youth is determinant of cardiovascular health in adulthood.*European Journal of Preventive Cardiology*, 2024.DOI:10.1093/eurjpc/zwae033. Doi: 10.1093/eurjpc/zwae033.

6. Gary CH, Gan BS, MBBS,Krishna K, Kadappu MBBS, et al.Exercise E/e′ is a determinant of exercise capacity and adverse cardiovascular outcomes in chronic kidney disease - sciencedirect.*JACC: Cardiovascular Imaging*, 2020, 13(12):2485-2494.Doi:10.1016/j.jcmg.2020.05.044.

7. Sayan M. The cumulative effect of insufficient sleep on adolescent obesity: evidence from a Spanish cohort. *European Journal of Preventive Cardiology.*2023.Doi:10.1093/eurjpc/zwad175.

8. Ozturk B, Duruturk N. Effect of telerehabilitation applied during COVID-19 isolation period on physical fitness and quality of life in overweight and obese individuals. *International Journal of Obesity.*2022; (1):46.Doi: 10.1038/s41366-021-00965-5.

9. Ho C C, Lee P F, Chen H L,et al. Poor health-related physical fitness performance increases the overweight and obesity risk in elderly people from Taiwan. 2020.Doi:10.21203/rs.3.rs-45860/v2.

10. Rozanski A, Blumenthal J A, Hinderliter A L, et al. Cardiology and lifestyle medicine.*Progress in Cardiovascular Diseases*, 2023; 77: 4-13. Doi: 10.1016/j.pcad.2023.04.004.

11. Su J, Jiang Y, Fan X, Tao R, Wu M, Lu Y, et al. Association between physical activity and cancer risk among Chinese adults: a 10-year prospective study. *The international journal of behavioral nutrition and physical activity.*2022; 19(1):150. Doi: 10.1186/s12966-022-01390-1.

12. [Health Care Index by Country 2024 (numbeo.com)](https://www.numbeo.com/health-care/rankings_by_country.jsp).

13.Fadillah AN, Maulang I, Hardiyanty N. The correlation between sedentary lifestyle and physical fitness level in adolescents. *Enfermería Clínica.* 2021; 31: S668-671.

14. Cantarero AB, LÓPEZ MS, Bueno CA, Vizcaino VM. Association between physical activity, fitness, sedentary lifestyle and health related quality of life in healthy children and adolescents: a protocol for a systematic review.PROSPERO 2015 CRD42015025823 Available from: https://www.crd.york.ac.uk/prospero/display_record.php?ID=CRD42015025823.

15. Jopkiewicz A, Gawron J, Nowak SA. Physical activity and fitness of adults aged 20–59 years. *Human Movement.*2015; 16(3).Doi: <https://doi.org/10.1515/humo-2015-0037>.

16. Klockziem Y, Tara T. Effect of a lifetime health and fitness class on college students. 2015, *Walden Dissertations and Doctoral Studies*. 1559. https://scholarworks.waldenu.edu/dissertations/1559.

17. Wang M. Physical activity and fitness of children and adolescents aged 6-19 in China: Current status and trends [C]//Compilation of Abstract of Papers at the 10th National Sports Science Conference in 2015 (Part 1). 2015.

18. Yan X, Pei H, Zhang Y, Kang N, Chen G, Wang D. Association between physical activity and physical fitness among adults aged 40 to 79 years—Beijing,China,2020–2021. *Chinese Center for Disease Control and Prevention Weekly Report(English)*.2022; 4(42): 936-940.

19. Tian Y, Jiang C, Wang M, Cai R, Zhang Y, Zhongtao H, et al. Body mass index, leisure-time physical activity and physical fitness in Chinese adults: a series of national surveys from 2000 to 2014. *The Lancet Diabetes & Endocrinology*, 2016; 4(6): 487–497. https://doi.org/10.1016/S2213-8587(16)00081-4.

20. Bi C, Lin H, Zhang J, Gui X, Shi Z. Regional variation in growth status: A cross-sectional survey among Tibetan adolescents living at three different high altitudes in Tibet, China. *American journal of human biology: the official journal of the Human Biology Council.*2022;e23856.<https://doi.org/10.1002/ajhb.23856>.

21. Zhang F, Bi C, Yang J, Zhang X, Song Y, Liu Y, et al. The sex-based disparity in BMI-for-age z-score trends among Xinjiang children and adolescents using four rounds of cross-sectional surveys from 1985 to 2014. *Journal of Public Health.*2020; 42(4):731-739.Doi：10.1093/pubmed/fdz189.

22. Liu S. Research on the construction of fitness courses in colleges and universities from the perspective of lifelong physical exercise. *Journal of Higher Education Research.*2022; 3(4):380-384.Doi：10.32629/jher.v3i4.1017.

23. Voelcker-Rehage C. Benefits of physical activity and fitness for lifelong cognitive and motor development—Brain and Behavior. *Sports and Exercise Psychology Research*. 2016;43-73.Doi: 10.1016/B978-0-12-803634-1.00003-0.

24. Hsu HC, Liang JM, Chu IH, Chang JJ, Wu WL. The influence of a new intervention model of community-based physical fitness exercise on the older adults' adherence to physical activity and the improvement of functional fitness. *Topics in Geriatric Rehabilitation.*2022;2(38): 140-148.Doi：10.1097/TGR.0000000000000355.

25. Yuan J M, Sun F, Zhao X, et al. The relationship between mindfulness and mental health among Chinese college students during the closed-loop management of the COVID-19 pandemic: *A moderated mediation model. Journal of Affective Disorders.* 2023; 327:137-144.Doi:10.1016/j.jad.2023.02.012.

26. Alhaleeq M, Zimi'A AA. Establishing Norms for some of the physical fitness components for undergraduate physical education students at the Hashemite university. *Advances in Physical Education.*2012; 05(3):170-175.Doi：10.4236/ape.2015.53021.

27. Qian S. Application of college physical education teaching strategies from the perspective of lifelong fitness. *Journal of Jiangxi Electric Power Vocational and Technical College.*2022; 35(3):33-34.

28. Yin XJ, Ji L, Zhang ZG, Ji CY, Du JQ. Research on the trend in physical health of the Chinese college students. *Journal of Beijing sport university*. 2012; 35(09):79-84.Doi：CNKI:SUN:BJTD.0.2012-09-019.

29. Zhang F, Yin X, Bi C, Ji L, Wu H, Li Y, et al. Psychological symptoms are associated with screen and exercise time: a cross-sectional study of Chinese adolescents. *BMC Public Health.*2020; 20(1):1695.Doi：10.1186/s12889-020-09819-7.

30. Bi C, Lin H, Zhang J, Zhao Z. Association between sleep quality and depression symptoms in Chinese college students during the COVID-19 lockdown period. *Children.*2022; 9(8).Doi: [10.3390/children9081237](https://doi.org/10.3390/children9081237).

31. Kidokoro T, Tomkinson GR, Jlang J, Suzuki K. Physical fitness before and during the COVID-19 pandemic: Results of annual national physical fitness surveillance among 16,647,699 Japanese children and adolescents between 2013 and 2021. *Sports and Health Sciences (English).*2023; 12(2):246-254.

32. Cao H.Analysis and countermeasure research on students’ physical health test results of Xi’an shiyou university. *Journal of Mudanjiang Normal university (Natural sciences edition)*. 2019; (04):61-64.Doi：CNKI:SUN:MDJZ.0.2019-04-018.

33. Song YM. A survey on the physical fitness status of male students in different grades at Huainan Normal University. *Industry and Technology Forum.* 2017;16(7): 2.Doi：10.3969/j.issn.1673-5641.2017.07.063.

34. Hu RC, Peng E. Ananlysis on the difference of physical fitness testing scores of inner mongolia undergraduate students--based on the results from inner mongolia agricultural university. *Journal of Inner Mongonlia Normal University (Natural Science Edition)*. 2016; 45(06):889-891.

35. Miao Y. An analysis of the factors affecting the innovation ability of sports college students——illustrated by the case of Shanghai University of Sport. *Contemporary Sports Technology.*2017; 7(19): 149-151. Doi: 10.16655/j.cnki.2095-2813. 2017.19.149.

36. Wang S. The analysis of factors affecting the physical fitness of college students from the perspective of physical education. *Journal of Xi'an Shiyou University(Social Science Edition).*2011; (4): 102-110.

37. Jin L, Li LI, Zhou Y, Zhou Y. How to remind people to work out via feedback: evidence from a field experim*ent. MPRA Paper.*2022. 112418, University Library of Munich,Germany.https://ideas.repec.org/p/pra/mprapa/112418.html.

38. Ferguson J: Establishing sports systems: A practical approach. *Open Journal of Social Sciences.*2022; 75-98.

39. Manninen M, Campbell S. The effect of the sport education model on basic needs, intrinsic motivation and prosocial attitudes: A systematic review and multilevel meta-analysis. *European Physical Education Review.*2022; 28(1):78-99.Doi：10.1177/1356336X211017938.

40. Wu X. Promoting nursing development towards health for all:Overview and prospect of nursing development in China. *International Nursing Science (English).*2023; 10(1):4.

41. Luo H, Zhang Q, Niu Y, Kan H, Chen R. Fine particulate matter and cardiorespiratory health in China:A systematic review and meta-analysis of epidemiological studies. *Journal of Environmental Science: English Edition.*2023; (1):306-316.

42. Wang ZS. A study on the differences in physical health issues among college students of different majors - Taking Lvliang College as an example. *Boxing and Fighting*. 2017;(2X):1.

43. Shen RN, Chen LQ. A comparative study on physical activities and health fitness of different major female graduate students. *Liaoning Sport Science and Technology*.2015; 37(05):55-58.Doi:10.13940/j.cnki.lntykj.2015.05.019.

44. Wang W. Research on the status Quo of college students' physical health and its influencing factors -- taking Nanchang Hangkong University as an example. *Master thesis of Nanchang Hangkong University*. 2016.

45. Lv KW. A study on the correlation between lifestyle and physical fitness of college students. *Master thesis of Taiyuan University of Technology*. 2022.

46. Liang J. Comparative analysis of Xinyu University students in different grades of physical health research. *Academic Exchange Conference on "National Fitness, Scientific Sports*". 2016.

47. Ping Y. The comparative analysis of the education of the art and physics major in PE. *Master thesis of Shanghai Normal University*. 2008.

48. Zhang H, Xu Y, Gao FY, Zhang B. Investigation on physical health status of college students in Tongling,Anhui. *Journal of Qiqihar Medical University*. 2018; 39(2):202-204.

49. Sun QR. Analysis of the current situation of dietary nutrition and physical activity of college students in Jiangsu province: A preliminary study using GIS. *Master thesis of Nanjing Sport Institute*. 2019.

50. Bi CJ, Yang JM, Sun J, Yi S, Wu XY, Zhang F. Benefits of normal body mass index on physical fitness A cross-sectional study among children and adolescents in Xinjiang Uyghur Autonomous Region, China. *PLOS ONE.*2019; 14(8):1-12.<https://doi.org/10.1371/journal.pone.0220863>.

51. Yang M, Sun J, Yan G, Lou X, Peng Y. Relevance between BMI and fitness quality index of students. *Chinese Journal of School Health.*2018; 39(05):688-690+693.Doi:10.16835/j.cnki.1000-9817.2018.05.014.

52. Jiang Y, Ding C, Shen B, Zhao S. The relationship between physical fitness and BMI among Chinese University students: results of a Longitudinal study. In: *CIPAE 2021: 2021 2nd International Conference on Computers, Information Processing and Advanced Education.* 2021;Doi:10.1145/3456887.3456934.

53. Bi CJ, Zhang F, Gu Y, Song Y, Cai X. Secular trend in the physical fitness of Xinjiang children and ad*olescents between 1985 and 2014. International journal of environmental research and public health.* 2020; 17(7): 2195; <https://doi.org/10.3390/ijerph17072195>.

54. Flores Navarro-Perez C, Fernandez-Aparicio A, Gonzalez-Jimenez E,et al. Effects of COVID-19 lockdown on the dietary habits and lifestyle in a population in southern Spain: a cross-sectional questionnaire. *European journal of clinical nutrition*. 2022; 6(76): 883-890.Doi：10.1038/s41430-021-01034-w.

55. Xu BR. Study on the current situation and correlation of physical activity level,sedentary behavior and physical health of primary and middle school students in Nanjing ——Taking some primary and middle school students in

Nanjing as an example. *Master thesis of Nanjing Sport Institute.* 2022.

56. Fountaine CJ, Liguori GA, Mozumdar A, Jr JMS. Physical activity and screen time sedentary behaviors in college students. *Dissertations & Theses Gradworks*. 2011;(2): 101-111. Doi:http://dx.doi.org/.
