# Supplementary material for: A comparative study on the physical fitness of college students from different grades and majors in Jiangxi province
Source: PLoS One. 2024 Oct 16;19(10):e0308483. doi: 10.1371/journal.pone.0308483 (PMC11482695; doi:10.1371/journal.pone.0308483)
Supplement: Attachment — Submitted filename: Response to Reviewers.docx [file pone.0308483.s005.docx]

PONE-D-23-32339

A comparative study on the physical fitness of college students from different grades and majors in Jiangxi province

PLOS ONE

1) "For example, among all majors, girls majoring in art tended to be slimmer. This high probability may be related to the difference between liberal humanities and science courses. There are more experimental courses in science and far more opportunities for exercise, while there are more liberal arts and history courses in humanities with longer sedentary hours"

This statement contradicts itself - it is saying girls majoring in art tend to be slimmer; but then says those in liberal arts courses have longer sedentary hours.

Response: Deleted the following sentence, "For example, among all majors, girls majoring in art tended to be slimmer" , to avoid ambiguity.

2) "Parents or students themselves do not attach importance to fitness."

This statement is not supported by evidence. Please provide the evidence or remove it.

Response: Deleted the following sentence, "Parents or students themselves do not attach importance to fitness."

3) " Constructing a healthy China requires the support of people's good exercise habits, especially for college students who are valuable talent and the future of our motherland."

Please rephrase the use of "motherland".

Response: Replaced "our motherland" with "China."
